# Changes in neural responses during affective and non-affective tasks and improvement of posttraumatic stress disorder symptoms following trauma-focused psychotherapy

**DOI:** 10.1038/s41398-023-02375-9

**Published:** 2023-03-09

**Authors:** Mayuresh S. Korgaonkar, Kim L. Felmingham, Gin S. Malhi, Thomas H. Williamson, Leanne M. Williams, Richard A. Bryant

**Affiliations:** 1 Brain Dynamics Centre, Westmead Institute for Medical Research, The University of Sydney, Westmead, Australia; 2 Department of Psychiatry, University of Sydney, Westmead, Australia; 3 Discipline of Psychological Science, University of Melbourne, Melbourne, Australia; 4 School of Psychology, University of New South Wales, Kensington, Australia; 5 Department of Psychiatry and Behavioral Sciences, Stanford University, Stanford, USA; 6 Sierra-Pacific Mental Illness Research, Education, and Clinical Center (MIRECC) VA Palo Alto Health Care System, Palo Alto, USA

**Keywords:** Psychiatric disorders, Human behaviour

## Abstract

At least one-third posttraumatic stress disorder (PTSD) patients do not respond to trauma-focused psychotherapy (TF-psychotherapy), which is the treatment of choice for PTSD. To clarify the change mechanisms that may be associated with treatment response, this study examined changes in neural activations during affective and non-affective processing that occur with improvement of symptoms after TF-psychotherapy. This study assessed PTSD treatment-seeking patients (*n* = 27) prior to and after TF-psychotherapy using functional magnetic resonance imaging when they completed three tasks: (a) passive viewing of affective faces, (b) cognitive reappraisal of negative images, and (c) non-affective response inhibition. Patients then underwent 9 sessions of TF-psychotherapy, and were assessed on the Clinician-Administered PTSD Scale following treatment. Changes in neural responses in affect and cognitive processing regions-of-interest for each task were correlated with reduction of PTSD severity from pretreatment to posttreatment in the PTSD cohort. Data from 21 healthy controls was used for comparison. Improvement of symptoms in PTSD were associated with increased activation of left anterior insula, reductions in the left hippocampus and right posterior insula during viewing of supraliminally presented affective images, and reduced connectivity between the left hippocampus with the left amygdala and rostral anterior cingulate. Treatment response was also associated with reduced activation in the left dorsolateral prefrontal cortex during reappraisal of negative images. There were no associations between response and activation change during response inhibition. This pattern of findings indicates that improvement of PTSD symptoms following TF-psychotherapy is associated with changes in affective rather than non-affective processes. These findings accord with prevailing models that TF-psychotherapy promotes engagement and mastery of affective stimuli.

**Clinical Trials Registration:** Trial Registration: Prospectively registered at Australian and New Zealand Clinical Trials Registry, ACTRN12612000185864 and ACTRN12609000324213. https://www.anzctr.org.au/Trial/Registration/TrialReview.aspx?id=83857

## Introduction

Trauma-focused psychotherapy (TF-psychotherapy) is the frontline treatment of choice for posttraumatic stress disorder, and is recommended in most treatment guidelines for PTSD [1]. TF-psychotherapy is an umbrella term that refers to a collection of psychological treatments that involve emotional processing of trauma memories, and to varying degrees also place emphasis on addressing maladaptive appraisals about the trauma. Common variants of TF-psychotherapy include Prolonged Exposure, Cognitive Processing Therapy, Eye Movement Desensitization and Reprocessing, Narrative Exposure Therapy, Cognitive Therapy, and Brief Eclectic Therapy. Although TF-psychotherapy is the treatment of choice for PTSD, up to one-half of patients do not respond to this treatment [2, 3]. This sub-optimal response rate points to the need to understand the underlying change mechanisms associated with improvement with TF-psychotherapy in order to identify potential factors that may contribute to better outcomes.

One of important lines of enquiry to study mechanisms of change in TF-psychotherapy is understand how neural functioning is associated with symptom improvement. Numerous studies of adults with PTSD have used functional magnetic resonance imaging (fMRI) during a range of tasks to map how treatment response is associated with neural changes when performing different tasks. Most studies have employed affective tasks that include fear conditioning [4], affective Stroop task [5, 6], viewing [7–10] or regulating [11] affective or trauma-related stimuli, anticipation of affective stimuli [12, 13], and social judgements [14]. Only one study has employed a fully non-affective task, which included a response inhibition paradigm [15]. One recent systematic review of these studies concluded that these studies converge on the finding that better treatment response is associated with increased activation of prefrontal cortical regions [16], although it should be noted that not all studies have found this. This activation of the prefrontal cortex is consistent with major models of PTSD that posit that TF-psychotherapy involves extinction processes in which repeated exposure to memories or reminders of the trauma in a safe context inhibit prior associations of fear with these stimuli [17]. Extinction has been linked to recruitment of prefrontal cortical regions in both human and animal studies [18]. Interestingly, there are mixed findings regarding amygdala activation and treatment response, which would be predicted by extinction models with most studies reporting no changes in amygdala activation following treatment [16]. It is notable that nearly all fMRI studies that have investigated the association between treatment outcome and neural activation have employed affective tasks. One study that used a response inhibition task found no association between treatment response and neural activation change [15].

There are no previous studies that have employed both affective and non-affective paradigms in the same treatment study to investigate how treatment response is associated with changes in neural functioning following TF-psychotherapy in PTSD. The goal of the present study was to examine the relationship between response to TF-psychotherapy in adults with PTSD with changes in neural activity and connectivity across affective and non-affective tasks. In this sense, this study attempted to extend on most previous studies by utilizing diverse paradigms to provide a more comprehensive investigation of neural functions that may be affected by TF-psychotherapy. This study focused on passive viewing of affective stimuli, reappraisal of traumatic stimuli, and response inhibition of neutral stimuli to provide indices of distinct neural functions in the context of treatment response. We hypothesized that improvement following TF-psychotherapy will be associated with an increase in activation of the prefrontal cortical regions for both the passive viewing and reappraisal affective paradigms and this activation to be at levels expected in healthy individuals post treatment. Due to only one previous treatment study in non-affective paradigms, we tested whether the prefrontal cortical activation for response inhibition also significantly increases following TF-psychotherapy. Due to mixed previous results for the amygdala, we did not have a particular hypothesis for changes in amygdala activation for the two affective paradigms.

## Materials and methods

### Participants

Participant recruitment for the study commenced from August 2009 and ended June 2014. Participants were 51 treatment-seeking patients, 36 of whom had viable imaging data at baseline and 27 of these with follow-up MRI data. For this manuscript we include data from the 27 PTSD patients (13 females; age = 40.9 ± 11.8 years) who completed MRIs at both visits (see CONSORT diagram in Supplementary Fig. S1).

The PTSD sample had developed PTSD after experiencing assault, childhood abuse, motor vehicle accidents, or during police duties. PTSD was diagnosed according to DSM-IV criteria by masters or doctoral level clinical psychologists using the Clinician Administered PTSD Scale (CAPS; [19]. Symptoms were detected according to the ‘2/1’ method, in which symptoms were experienced for at least twice a month and caused moderate levels of distress. Levels of depression and anxiety were assessed using the Depression, Anxiety, and Stress Scale (DASS). Patients that reported psychosis, bipolar disorder, substance dependence, neurological disorders, or moderate to severe brain injury were excluded from the study. Patients taking medication (10 on selective serotonin reuptake inhibitors) were included on the condition that the dosage was stable for the previous two months and continued to be stable for the duration of the study. In addition, 21 controls (9 females; mean age 36.9 ± 14.0 years) who were age and gender-matched to the PTSD group were included in the study. Control participants had not experienced a Criterion A stressor and did not have an Axis I disorder, as assessed by the Mini International Neuropsychiatric Interview (MINI version 5.5) [20].

All participants underwent clinical and imaging assessments at baseline and again 12 weeks later. This study was approved by the Western Sydney Area Health Service Human Research Ethics Committee and informed consent was obtained from participants.

### Treatment protocol

Approximately two weeks after baseline clinical and fMRI testing, participants in the PTSD group began a 9-week TF-psychotherapy treatment protocol administered by a doctoral or masters-level clinical psychologist. Treatment occurred in weekly 60–90 min sessions and was consistent with TF- psychotherapy protocols. Therapy comprised one session of psychoeducation about psychological responses to trauma, followed by six sessions of 40 min imaginal exposure to the trauma memory, together with instructions regarding in vivo exposure to avoided situations. In these sessions cognitive reframing of catastrophic thoughts about the trauma and oneself were addressed, as well as an additional session that reinforced cognitive reframing exercises. A final session instructed participants on relapse prevention strategies [21, 22]. This therapy procedure represents standard TF-psychotherapy procedures [23]. Independent clinical psychologists rated the fidelity of 130 sessions (18%), which indicated full adherence to the treatment protocols and high level of quality on a 7-point scale (*M* = 6.11 ± 1.32). A posttreatment assessment was conducted by an independent clinical psychologist one week following completion of the course of treatment using the CAPS. Changes in PTSD symptom severity was calculated as follows and used for analyses: first, post-treatment CAPS scores were subtracted from pre-treatment scores, then the result of this was divided by the pre-treatment CAPS score to create a measure of change that was independent of initial symptom severity. A higher score on this measure corresponds to greater improvement in PTSD symptoms.

### Imaging acquisition and data analyses

Functional brain images were collected with a 3.0 T GE Signa HDx scanner (GE Healthcare, Milwaukee, Wisconsin) using an echo planar imaging protocol and an eight-channel head coil and were analyzed using SPM12 (Statistical Parametric Mapping) software on MATLAB2018. Details of MRI acquisition, tasks and pre-processing steps of all fMRI data has been described in detail previously [24–28] and is also provided in the supplementary section (S1).

**Fig. 1:**
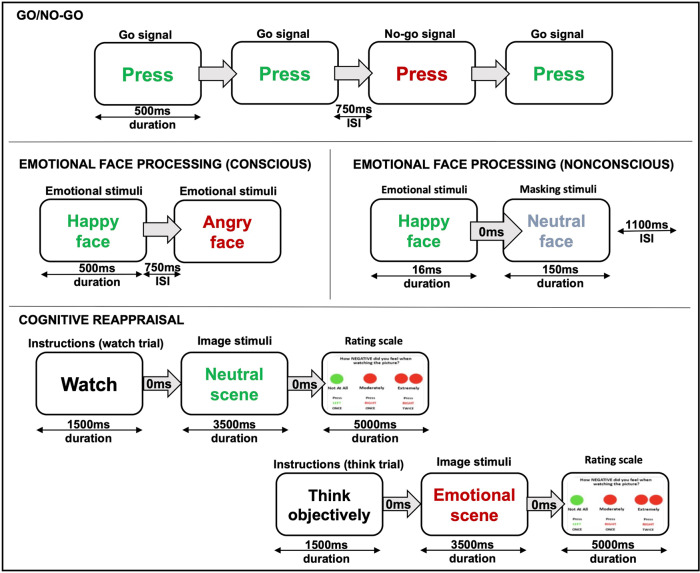
Summary of tasks performed by participants. Each participant performed every task. There was a conscious and nonconscious version of the emotional faces task.

Participants completed the following fMRI tasks (Fig. 1): a) *Go/No-Go Task:* a non-affective response inhibition paradigm where participants were instructed to button press to Go trials (word “PRESS” in green writing; 180 trials) and refrain pressing on the No-Go trials (“PRESS” in red; 60 trials) presented in pseudorandom order; b) *Emotional Face Processing Task (conscious and non-conscious runs):* passive viewing of faces with angry, fearful, happy, sad, disgust or neutral expressions (each emotion presented in 10 s blocks of 8 faces and repeated five times in random order; each face for 500 ms with 750 ms interstimulus interval). In the nonconscious run, each emotional face was presented for 16.7 ms, immediately followed by a neutral face for 150 ms (backward masking so that participants are not consciously aware of the presented emotion), and then a 1083.3 ms interstimulus interval; c) *Cognitive Reappraisal Task:* emotion regulation paradigm where participants either reappraised (down-regulation of emotional response) or watched traumatic and neutral stimuli (three blocks [Think, Neutral, and Watch] of ten images each; each image presented for 10 s including a rating scale of how negative the image made them feel).

First level general linear models were performed for each task and contrast images generated for each task for every participant. We then employed a region of interest (ROI) analysis approach for second level analyses. For the emotional face processing task, we selected ROIs based on networks involved in positive and negative emotion processing [29]. We examined regions comprising the negative affect network: subgenual and pregenual anterior cingulate cortices (sgACC, pgACC), bilateral amygdala, insula, and hippocampus. We also examined the medial prefrontal cortex (dmPFC) and the bilateral dorsolateral prefrontal cortex (dlPFC) as evidence from a meta-analysis indicates these regions are functionally connected to the negative affect network and have some role in emotional processing and regulation [30]. The amygdala, hippocampus, insula ROIs were defined using the AAL atlas, whereas the remaining ROIs (dlPFC, dmPFC, sgACC, pgACC) were defined based on coordinates derived from previous meta-analyses and used in our previous published work using these tasks. Details of all ROIs are provided in the supplementary (Supplementary Table S1 and Supplementary Fig. S2). For the cognitive reappraisal task, we examined the dmPFC, bilateral dlPFC and bilateral amygdala, selected based on our previous evidence of regions that are engaged using this task and can be predictive of TF-psychotherapy outcome [27]. In the Go/No-Go task, we examined the cognitive control network (bilateral dlPFC, dorsal ACC [dACC], inferior and superior parietal cortices), based on previous studies indicating its role in the association of response inhibition and treatment response [31, 32]. All statistical analyses were conducted voxel-wise using a family-wise error corrected *p*-value of 0.05 (*pFWE* < 0.05). Multiple comparisons were controlled by incorporating all task-specific ROIs into a single mask used for the relevant task.

As done in our previous work using the 3 tasks, contrast images reflecting response inhibition in the Go/No-Go task were created by comparing No-Go trials to Go trials and to the implicit baseline. Contrast images for the emotional faces processing tasks (conscious and nonconscious) were derived by comparing each affective expression (angry, fearful, happy, sad, disgust) to the neutral condition or to the implicit baseline and were entered as a within-subjects factor in a repeated-measures design. For the reappraisal task, the contrast image for cognitive reappraisal was created by comparing Think vs Watch trials, and the contrast image for emotional reactivity was created by comparing Watch trials to Neutral trials.

To evaluate activation changes over the course of therapy, each participant’s baseline contrast image was subtracted from their post-treatment contrast image. This produced a ‘delta’ contrast image which reflected the degree of change in activation between the two scans which was then used to evaluate voxel-wise correlations with each participant’s CAPS-change score to identify brain regions associated with treatment response (at *pFWE* < 0.05).

For functional connectivity analyses, a generalized psychophysiological interaction (gPPI; [33] was conducted to evaluate associations between functional connectivity changes and treatment response. ROIs displaying significant results in the activation analyses were selected as seed regions, and the processed delta contrast images were used to evaluate voxel-wise correlations with treatment response (at *pFWE* < 0.05) using the same task-specific ROI mask used in the activation analyses. For each task, the gPPI model included a psychological regressor for onset times for each task condition, a physiological regressor (time series of the seed region), and psychophysiological regressors of the interaction between each condition and the time series regressor. The psychophysiological term against the time course of other brain regions evaluates task-related connectivity.

To compare the treatment related neural changes observed in the PTSD group with controls, we performed a voxelwise linear mixed model analysis using a MATLAB based toolbox called *Sandwich Editor* (SwE). This was conducted on ROIs displaying significant results in the second level analyses for each fMRI task. The SwE toolbox allows accounting for all possible random effects by using an unstructured covariance correlation structure and is robust for longitudinal analyses of neuroimaging data [34]. Time of scan (pre-treatment or post-treatment) and task condition were entered as within-subjects factors and group (PTSD or control) was entered as a between-subjects factor. For the emotional face processing tasks, emotion was also entered as a within-subjects factor. Contrasts were conducted to detect group-by-time interactions at *pFWE* < 0.05.

## Results

### Clinical outcomes

All participants in the patients group met the DSM-IV criteria for PTSD prior to treatment and completed 7-9 sessions of treatment. The mean CAPS score before treatment was 73.3 ± 18.1 which reduced to 26.3 ± 19.4 post-treatment. Nineteen (70.4%) participants had their symptoms reduce by more than 50% (treatment responders). PTSD participants reported markedly higher scores on the DASS Depression, Anxiety, and Stress scales (*p* < 0.001). PTSD and control participants did not differ in age, gender, or education level (see Table 1).

**Table 1:** Participant Characteristics.

	PTSD	Controls	*P*-Value	Responders	Nonresponders
	(*n* = 27)	(*n* = 21)		(*n* = 19)	(*n* = 8)
**Age, mean (SD)**	40.9 (11.8)	36.9 (14.0)	0.119	40.4 (13.4)	41.9 (7.2)
**Sex,** ***n*** **(%)**					
Male	14 (52%)	9 (42.9%)	0.743	10 (53%)	4 (50%)
Female	13 (48%)	12 (57%)		9 (47.4%)	4 (40%)
**Years of Education, mean (SD)**	14.4 (3)	15.5 (2.6)	0.164	14.1 (2.9)	15.1 (3.4)
**DASS Scores, mean (SD)**					
DASS Depression	22.0 (9.5)	3.3 (5.7)	< 0.001	22.3 (10.6)	21.1 (6.8)
DASS Anxiety	15.6 (9.5)	2.3 (4.4)	< 0.001	14.4 (10.2)	20.9 (4.19)
DASS Stress	24.2 (8.0)	6.4 (7.4)	< 0.001	22.9 (8.4)	27.1 (6.5)
**CAPS Scores, mean (SD)**					
Baseline CAPS severity	73.3 (18.1)	-		75.6 (19.6)‘	67.8 (13.7)
Posttreatment CAPS severity	26.3 (19.4)	-		16.6 (11.8)	49.1 (14.0)
**Time since Trauma, months, mean (SD)**	18.5 (15.0)	-		19.8 (16.9)	15.3 (9.2)
**Type of trauma,** ***n*** **(%)**		-			
Childhood abuse	3 (11%)	-		3 (16%)	0 (0%)
Motor vehicle accident	6 (22%)	-		4 (21%)	2 (25%)
Police-related trauma	8 (30%)	-		5 (26%)	3 (38%)
Assault	10 (37%)	-		7 (37%)	3 (38%)
**Prescribed SSRI,** ***n*** **(%)**	10 (37%)	-		7 (37%)	3 (38%)
**Comorbidities,** ***n*** **(%)**					
Major Depressive Disorder	11 (44%)	-		6 (32%)	5 (63%)
Social Phobia	1 (4%)	-		0 (0%)	1 (13%)
Panic Disorder	4 (15%)	-		3 (16%)	1 (13%)
Agoraphobia	8 (30%)	-		7 (37%)	1 (13%)
Generalised anxiety disorder	6 (22%)	-		4 (21%)	2 (25%)
Obsessive compulsive disorder	1 (4%)	-		1 (5%)	0 (0%)

*CAPS* Clinician Administered PTSD Scale, *DASS* Depression Anxiety Stress scale, *SD* Standard deviation, *SSRI* Selective serotonin reuptake inhibitor.

### Go/No-go task

There were no significant correlations between change in activation for either of the cognitive control ROIs and CAPS-change scores for both response inhibition contrasts (NoGo-vs-Go and NoGo-vs-baseline).

### Emotional face processing task

**Fig. 2:**
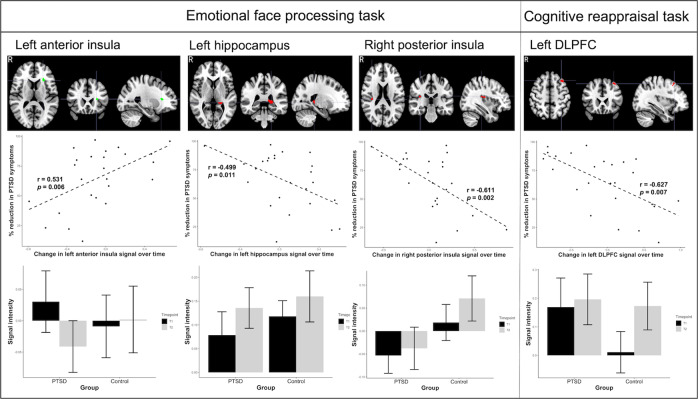
Changes in fMRI activation during the emotional face processing task and cognitive reappraisal task. Columns depict brain regions in which the change in activation during the task from pre-treatment (T1) to post-treatment (T2) significantly correlated with changes in PTSD symptoms. Scatter plots on the second row depict change in signal intensity in the identified region and percentage reduction in PTSD symptoms. Bar plots in the third row depict mean activation in these regions at T1 and T2 for the PTSD and control groups.

There were significant associations between activations and treatment response for the conscious and not for the non-conscious version of the emotion face processing task (Table 2 & Fig. 2). For the emotional face-vs-baseline contrast (main effect across emotions), increased activation in the anterior left insula from pre-treatment to post-treatment was associated with a reduction in PTSD symptoms (*pFWE* = 0.019). CAPS change scores also displayed a negative correlation with activity in both the left hippocampus (*pFWE* < 0.001) and posterior right insula (*pFWE* = 0.009), indicating that decreased activity in these regions over time was associated with improved PTSD symptoms. For the emotional face-vs-neutral face contrast, there were no significant correlations between CAPS change scores and changes in activation.

**Table 2:** Summary of significant associations of changes in neural activations and connectivity with reduction in PTSD symptoms.

Brain region	Direction of CAPS correlation	Peak MNI Coordinates (X, Y, Z)	Cluster size in voxels	Peak z-score	*p*-value (FWE)
*Neural Activation*					
*Go/No-Go task*					
-	NS	-	-	-	-
*Emotional Faces (Conscious) task*					
L Anterior Insula	Positive	−26, 26, 12	292	4.07	0.014
L Hippocampus	Negative	−18, −36, 6	715	5.1	0.003
R Posterior Insula	Negative	34, −26 18	292	4.26	0.009
*Emotional Faces (Unconscious) task*					
-	NS	-	-	-	-
*Cognitive Reappraisal task*					
L dlPFC	Negative	−38, 24, 56	162	4.51	0.036
*Neural Connectivity*					
*Go/No-Go task*					
*-*	NS	-	-	-	-
*Emotional Faces (Conscious) task*					
*Left Hippocampus seed*					
sgACC	Negative	−6, 20, −6	230	3.96	0.003
L Amygdala	Negative	−22, −4, −26	43	3.17	0.024
pgACC	Negative	−4, 48, 2	17	2.79	0.048
*Right Insula (Anterior) seed*					
L dlPFC	Negative	−60, 14, 22	137	4.31	0.01
*Emotional Faces (Unconscious) task*					
-	NS	-	-	-	-
*Cognitive Reappraisal task*					
-	NS	-	-	-	-

*CAPS* ClinicianF Administered PTSD Scale, *dlPFC* dorsolateral prefrontal cortex, *FWE* Family-wise error, *L* Left, *MNI* Montreal Neurological Institute, *NS* Not significant, *pgACC* pregenual anterior cingulate cortex, *sgACC* Subgenual anterior cingulate cortex.

**Fig. 3:**
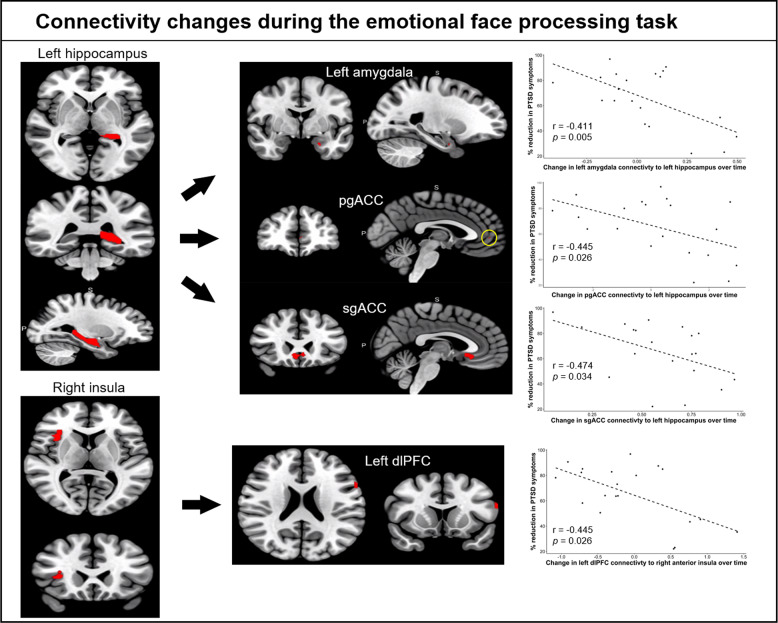
fMRI connectivity changes during the emotional face processing task. Changes in connectivity of the left hippocampus to the left amygdala, pgACC and sgACC, and of the right insula to the left dlPFC, significantly correlated with changes in PTSD symptoms.

ROIs displaying significant correlations with CAPS change scores were selected as seed regions for the connectivity (gPPI) analysis. The insula regions were split into anterior and posterior subregions (see supplementary section) because each pole correlated in opposite directions with CAPS change scores in the activation analyses. Using the left hippocampus as seed, significant negative correlations with CAPS change scores were found for the left amygdala (*pFWE* = 0.024), pgACC (*pFWE* = 0.048), and sgACC (*pFWE* = 0.003), indicating that decreased connectivity with these regions over time was associated with better treatment response (Fig. 3 & Table 2). Using the right anterior insula as seed, a significant negative correlation with CAPS change was found for the left dLPFC (*pFWE* = 0.01), indicating that decreased connectivity between these regions over time was associated with better treatment response. There were no significant connectivity relationships with CAPS change scores for the left anterior insula, left posterior insula, and right posterior insula seeds.

For the above significant effects, there were no significant differences in changes in activation or connectivity over time between the PTSD and control groups (no group-time interaction).

### Cognitive reappraisal Task

For the contrast reflecting cognitive reappraisal (Think vs Watch), there was a significant negative correlation between activity change in the left dlPFC and CAPS change scores (*pFWE* = 0.036) indicating that decreased activation from pre-treatment to post-treatment in this region was associated with improvement in PTSD symptoms (see Table 2). There were no significant correlations between CAPS-change scores and activation changes for the contrast reflecting emotional reactivity (Watch vs Neutral). There were also no significant correlations between changes in dlPFC connectivity and CAPS-change scores.

As for the emotion processing task, there were no differential effects for changes in left dlPFC activation over time between the PTSD and control groups.

## Discussion

It is notable that across the paradigms employed in this study, the two affective paradigms involved associations between improvement of PTSD symptoms after treatment and changes in neural activity. In contrast, the non-affective paradigm involving response inhibition was not implicated in any associations between symptom and neural changes. This latter finding accords with one previous study that also found no association between PTSD remission and neural activation during a Go-NoGo paradigm [15].

The finding that improvement after treatment was associated with decreased left hippocampal activation and connectivity when viewing affective faces accords with the role that the hippocampus plays in context processing. PTSD models have emphasized that the hippocampus is implicated in extinction memory because it enables differentiation between contexts and in this way can influence fear generalization [35]. This is particularly relevant with its connection with the amygdala and anterior cingulate cortex (which play a critical role in processing and regulation of emotions), which in our analysis were also found to be associated with improvement in symptoms (reduced connectivity over time). One meta-analysis has reported hyperactivation in the hippocampus in PTSD [36], although others have not [37, 38]. The current finding may suggest that successful remission of PTSD symptoms after TF-psychotherapy may involve more adaptive recruitment of context processing at a neural level.

The association between response and increased activation in the anterior insula as well as reduced activation in the posterior right insula during emotional processing can be understood in the context of the functional role of this region. The posterior insula encodes somatosensory information, which is integrated with affective information in the middle insula which projects to the anterior insula, in which limbic and sensory information is used for other cognitive processes [39, 40]. There are key connections between the posterior insula and subcortical regions implication in regulation of negative affect, such that some psychopathological disorders display greater activation of the posterior insula when viewing negative images [41, 42]. In the context of PTSD it is worth noting that decreased posterior insula recruitment is associated with parasympathetic activation [43], and this region has aberrant connectivity with key functional networks, including the salience network [44, 45]. The anterior insula is also considered a key component of the salience network and plays an important role in modulating threat reactivity via its connections to the cognitive control brain regions. This could explain our finding of reduced connectivity between anterior insula and the lateral frontal regions with improvement in treatment. Also a reduced connectivity between the anterior insula with frontal regions with treatment may mean reduction in excessive threat vigilance and so that these regions are now ‘available’ for better modulation of traumatic stimuli.

The observation that response was associated with decreased recruitment of the left dlPFC during reappraisal was contrary to our hypothesis and previous meta-analytical finding that prefrontal cortex activation is increased following treatment [16]. However as noted by this meta-analytical study, not all studies found an increase in prefrontal activation to be associated with symptom improvement. Nevertheless the involvement of the dLPFC in treatment response can be understood in the context of much evidence that reappraisal implicates prefrontal regions which purportedly down-regulate affective responses [46]. The dlPFC is part of a prefrontal network that is implicated in a range of cognitive and control functions [47]. It has connections to the anterior cingulate cortex, inferior frontal gyrus, caudate, and thalamus [48]. The role of the dlPFC in reappraisal is underscored by evidence that patients affected by lesions in this region are compromised in regulation of affect [49]. PTSD participants have greater functional connectivity in this region with a range of fronto-temporo-parietal networks, possibly reflecting compensatory effort to regulate emotional states [50]. TF-psychotherapy aims to promote down-regulation of aversive states, and the current treatment protocol engaged strategies directly intended to reappraise aversive states. To this end, the greater symptom reduction after TF-psychotherapy may be associated with reduced recruitment of this regulatory network as individuals become more efficient in their capacity to down-regulate.

We note several limitations in this study. First, the sample size available in our analysis was modest and further study is required with larger samples. Although we commenced with 37 PTSD participants, a significant number would not participate in a second scanning session, and future studies need to initially oversample in recruitment to ensure adequate sample size is maintained at the subsequent scanning session. Second, although we included a comparison healthy control group, our design lacked a wait-listed PTSD group that did not receive TF-psychotherapy. Even with our control group we did not observe differences in change in neural measures with treatment in PTSD. This could possibly be due to the limited sample size available. We conducted exploratory analyses after splitting PTSD responders and non-responders relative to controls at each time and observe differences in hippocampal and posterior insula activation at pre and post-treatment (supplementary section: Supplementary Figs. S3 & S4 and Supplementary Tables S2 & S3). However, the omission of a wait-list PTSD group precludes excluding the roles of time and natural remission as distinct from psychotherapy on changes in neural activations. Third, this study tested the association of neural activations with one variant of TF-psychotherapy, and there is a need to extend this line of research with other forms of psychotherapy and pharmacotherapy. Fourth, we did not investigate changes in neural activations and remission at longer-term follow-up, which would provide more enduring effects of therapy on neural processing. Fifth, our data was collected using an 8-channel head coil as this was the available hardware on our scanner at time of data acquisition. Newer coils such as 32 channel head coils with improved phased array systems have been demonstrated to improve measures obtained from fMRI scans and could have provided improved sensitivity to observe effects in our analysis [51]. Finally, although we correct for number of ROIs within each task in our analysis, we did not correct for possible type 1 effects due to inclusion of number of tasks and contrasts in our study. With this stringent correction only the finding of decrease in hippocampal activation and its connectivity with sgACC associated with improvement in symptoms during emotion face processing would survive significance.

In summary, this study demonstrates differential associations between affective and non-affective neural processing with level of remission of PTSD symptoms after TF-psychotherapy. This pattern of findings converges with the goals of TF-psychotherapy that enhances more effective and efficient mastery of affective states, which converges with extinction and cognitive models of PTSD that emotional processing as a central change mechanism of successful treatment.

## Supplementary information

Supplementary Section
