# Supplementary material for: Changes in neural responses during affective and non-affective tasks and improvement of posttraumatic stress disorder symptoms following trauma-focused psychotherapy
Source: Transl Psychiatry. 2023 Mar 9;13:85. doi: 10.1038/s41398-023-02375-9 (PMC9998447; doi:10.1038/s41398-023-02375-9)
Supplement: Supplementary file 1 — Supplementary Section [file 41398_2023_2375_MOESM1_ESM.docx]

**Supplementary Section**

Changes in Neural Responses During Affective and Non-Affective Tasks and Improvement of Posttraumatic Stress Disorder Symptoms Following Trauma-Focused Psychotherapy

Mayuresh S. Korgaonkar, PhD^1,2^, Kim Felmingham PhD^3^, Gin S. Malhi, MD^2^, Thomas H. Williamson^1,3^, Leanne M. Williams, PhD^5,6^, & Richard A. Bryant, PhD^1,3^

1. Brain Dynamics Centre, Westmead Institute for Medical Research, The University of Sydney.
2. Department of Psychiatry, University of Sydney
3. School of Psychology, University of New South Wales.
4. Discipline of Psychological Science, University of Melbourne
5. Department of Psychiatry and Behavioral Sciences, Stanford University
6. Sierra-Pacific Mental Illness Research, Education, and Clinical Center (MIRECC) VA Palo Alto Health Care System, Palo Alto

**Supplementary Figure Legend:**

**Supplementary Figure S1: CONSORT 2010 Flow Diagram.**

**Supplementary Figure S2: ROIs used in the study.**

**Supplementary Figure S3: Changes in fMRI activation for treatment responders and non-responders for neural measures that were significantly associated with change in symptoms in the main analysis.**

**Supplementary Figure S4: Changes in fMRI connectivity for treatment responders and non-responders for neural measures that were significantly associated with change in symptoms in the main analysis.**

**Supplementary Table Legend:**

**Supplementary Table S1: ROIs used in the study for each fMRI task.**

**Supplementary Table S2: Comparisons in activation between Responders, Non-Responders and Controls at the two timepoints.**

**Supplementary Table S3: Comparisons in connectivity between Responders, Non-Responders and Controls at the two timepoints.**

**Supplementary Figure S1: CONSORT 2010 Flow Diagram**

Healthy controls with follow-up MRIs (n = 21)

Analysed: PTSDs with follow-up MRIs (n = 27)

Allocated to TF-CBT (n= 84)

♦ Consented to MRI assessment (n = 51)

## Follow-Up

## Analysis

Not included in MRI study

Not included in MRI study

Lost to follow-up (n = 11)

Did not complete follow-up MRIs (n=13)

## Enrollment

## Allocation

Allocated to Control conditions (n= 72)

♦ Consented to MRI assessment (n = 0)

Randomized (n= 156)

Excluded (n= 48)

♦  Not meeting inclusion criteria (n= 28 )

♦  Declined to participate (n=8 )

♦  Other reasons (n=20 )

Assessed for eligibility (n= 204)

**S.1 fMRI data processing and Regions of Interest used in the Study**

*Imaging parameters*

Functional data was acquired with a 3.0T GE Signa HDx scanner (GE Healthcare, Milwaukee, Wisconsin) using an echo planar imaging protocol and an eight-channel head coil. 120 functional T2*-weighted volumes were acquired in each task run. These were comprised of 3.5mm 40 axial contiguous slices parallel to the intercommissural (AC-PC) line and a 2.5s TR, 27.5ms TE, 90° flip angle, 24x24cm^2^ FOV, and 64x64 matrix. At the start of the sequence, 3 dummy scans were acquired so that magnetization could stabilize to steady state. A high-resolution T1-weighted anatomical image was also acquired in the sagittal plane using a 3D spoiled gradient echo sequence with the following parameters: TR=8.3ms, TE=3.2ms, flip angle=11°, TI=500ms, NEX=1, ASSET=1.5, matrix size=256x256, 180 contiguous 1mm slices covering the whole brain resulting in an 1mm^3^ isotropic voxel resolution. This sequenced allowed for normalization of the functional images into standard space.

*Functional MRI Tasks*

Participants completed the following tasks during fMRI:

*1. Go/No-Go Task*

The Go/No-Go task is a non-affective paradigm which assesses response inhibition function. Participants were instructed to respond to Go trials (word “PRESS” displayed in green writing) by pressing a button as quickly as possible and refrain button pressing on the No-Go trials (“PRESS” in red writing). Stimuli were presented for 500ms with a 750ms interstimulus interval. There were 240 total stimuli – 180 Go trials and 60 No-Go trials presented in pseudorandom order. Performance on the task – number of commission errors (failure to withhold a response), number of omission errors (failure to respond on GO trials), and reaction time – was recorded for each participant.

*2. Emotional Face Processing Task*

Participants engaged in two passive viewing emotional face processing tasks. One task targeted the conscious processing of emotional stimuli and the other targeted nonconscious emotional processing. Both versions of this task are well-established (Bryant, Erlinger, Felmingham, Malhi, et al., 2020; Bryant et al., 2008; Korgaonkar et al., 2019; Korgaonkar, Grieve, Etkin, Koslow, & Williams, 2013) and have displayed predictive value for treatment outcomes of other clinical disorders (Williams et al., 2015). The task consisted of participants viewing a series of faces with angry, fearful, happy, sad, or neutral expressions. In the conscious version, each image was viewed for 500ms with a 750ms interstimulus interval. In the nonconscious version, the emotional face was presented for 16.7ms, immediately followed by a neutral face for 150ms, and then a 1083.3ms interstimulus interval. The neutral face backwardly masks the previous expression such that the participant is not consciously aware of the presented emotion. For both tasks, 240 total stimuli were grouped into blocks of 8 different faces of the same emotional expression. Each block lasted for 10s and each emotion was repeated 5 times in pseudorandom order. To control for potential priming effects, the nonconscious version of the task was conducted before the explicit version.

*3. Cognitive Reappraisal Task*

Participants completed a cognitive reappraisal task of traumatic stimuli used in prior neuroimaging studies to evaluate emotion regulation (Bryant, Erlinger, Felmingham, Klimova, et al., 2020; Ochsner, Bunge, Gross, & Gabrieli, 2002; Rai et al., 2021). The task consisted of two runs within a fMRI block design. Participants were presented with 20 negative stimuli (mean valence ratings, 2.18; mean arousal ratings, 6.91) and 20 neutral stimuli (mean valence ratings, 5.05; mean arousal ratings, 3.2), which were chosen from the IAPS image collection (Lang, 2005). For each run, images were presented in three blocks (Think, Neutral, and Watch) of ten images each, with each image presented for 10 seconds. The neutral stimuli were only used in the Neutral trials, whereas each negative image was used for the Think trials in one run and Watch trials in the other. A trial consisted of the word Think or Watch appearing on-screen for 1.5s, followed by the stimulus image for 5s, and finally a rating screen for 3.5s. Participants were instructed to rate how negative the image made them feel using a button box in the scanner which corresponded to a five-point scale (1=*not at all,* 5=*extremely*). In Think trials, participants were instructed to down-regulate emotional responses to distressing stimuli using cognitive reappraisal techniques (Goldin, McRae, Ramel, & Gross, 2008). For example, participants were asked to view stimuli from the perspective of a filmmaker and focus on technical details of the image. Prior to the fMRI session, cognitive reappraisal was explained to the participants with examples to ensure they understood the task. During Watch and Neutral trials, participants viewed the images without reappraising them. Blocks of Think and Watch trials were counterbalanced across participants (block order preserved across each participant’s two task runs) and were alternated with Neutral trials.

*Data pre-processing*

Pre-processing of all fMRI data (including realignment, normalization, and smoothing) was conducted using SPM12 (Statistical Parametric Mapping software). fMRI images were first realigned and unwarped to the initial image for the task run. The fMRI data was then normalized by using the FMRIB linear registration tool to co-register the functional data to the T1 scan and using the FMRIB nonlinear registration tool to normalize the T1 sequence to standard MNI space. A mask for the ventricles and white matter was used to discount the corresponding signal and correct any physiological noise. An 8mm Gaussian kernel was used to smooth the fMRI data. First-level analyses used a hemodynamic-response-convolved boxcar function to model the Blood Oxygen Level-Dependent (BOLD) response for each condition within the tasks (Go & NoGo condition for response inhibition; each emotion for the face processing task; and Think, Watch and Neutral for the cognitive reappraisal tasks). The resulting contrast images were normalized to 2mm^3^ isotropic standard space and then used for second-level analyses.

To identify scans with excessive movement or signal variations, we estimated 3 translational and 3 rotational motion parameters during realignment. A motion artifact was defined as a volume frame with greater than 0.5 m displacement from the previous frame in x, y, or z direction. An intensity artifact was defined as a volume with mean signal intensity that was greater than 3 standard deviations from the mean intensity over the entire scan. Problematic volumes were included as regressors in first-level analyses to discount to remove artifacts with scans with no participants with >25% such volumes.

Region of Interest Analyses:

**Supplementary Table S1: ROIs used in the study for each fMRI task.**

| **Region** | **Reference** | **Coordinates** | **Size** |
| --- | --- | --- | --- |
| *Emotional Face Processing Task* | |  |  |
| sgACC | Kober et al. 2008 | 0, 24, -8 | 10mm |
| pgACC | Kober et al. 2008 | 0, 42, 4 | 10mm |
| Left amygdala | AAL | - | - |
| Right amygdala | AAL | - | - |
| Left insula | AAL | - | - |
| Right insula | AAL | - | - |
| Left hippocampus | AAL | - | - |
| Right hippocampus | AAL | - | - |
| dmPFC | Kober et al. 2008 | 0, 18, 42 | 10mm |
| Left dlPFC 1 | Buhle et al. 2014, Berboth et al. 2021 | -36, 15, 57 | 10mm |
| Right dlPFC 1 | Buhle et al. 2014, Berboth et al. 2021 | 51, 15, 48 | 10mm |
| Left dlPFC 2 | Buhle et al. 2014, Berboth et al. 2021 | -51, 12, 21 | 10mm |
| Right dlPFC 2 | Buhle et al. 2014, Berboth et al. 2021 | 42, 30, 39 | 10mm |
| *Cognitive Reappraisal Task* | |  |  |
| Left amygdala | AAL | - | - |
| Right amygdala | AAL | - | - |
| dmPFC | Kober et al. 2008 | 0, 18, 42 | 10mm |
| Left dlPFC 1 | Buhle et al. 2014, Berboth et al. 2021 | -36, 15, 57 | 10mm |
| Right dlPFC 1 | Buhle et al. 2014, Berboth et al. 2021 | 51, 15, 48 | 10mm |
| Left dlPFC 2 | Buhle et al. 2014, Berboth et al. 2021 | -51, 12, 21 | 10mm |
| Right dlPFC 2 | Buhle et al. 2014, Berboth et al. 2021 | 42, 30, 39 | 10mm |
| *Go/No-go Task* |  |  |  |
| Left inferior parietal | Niendam et al. 2012 | -38, -52, 40 | 10mm |
| Right inferior parietal | Niendam et al. 2012 | 38, -50, 42 | 10mm |
| Left superior parietal | Niendam et al. 2012 | -28, -60, 44 | 10mm |
| Right superior parietal | Niendam et al. 2012 | 28, -60, 44 | 10mm |
| dACC | Niendam et al. 2012 | 0, 16, 40 | 10mm |
| Left dlPFC 1 | Buhle et al. 2014, Berboth et al. 2021 | -36, 15, 57 | 10mm |
| Right dlPFC 1 | Buhle et al. 2014, Berboth et al. 2021 | 51, 15, 48 | 10mm |
| Left dlPFC 2 | Buhle et al. 2014, Berboth et al. 2021 | -51, 12, 21 | 10mm |
| Right dlPFC 2 | Buhle et al. 2014, Berboth et al. 2021 | 42, 30, 39 | 10mm |

**Supplementary Figure S2: ROIs used in the study**

**
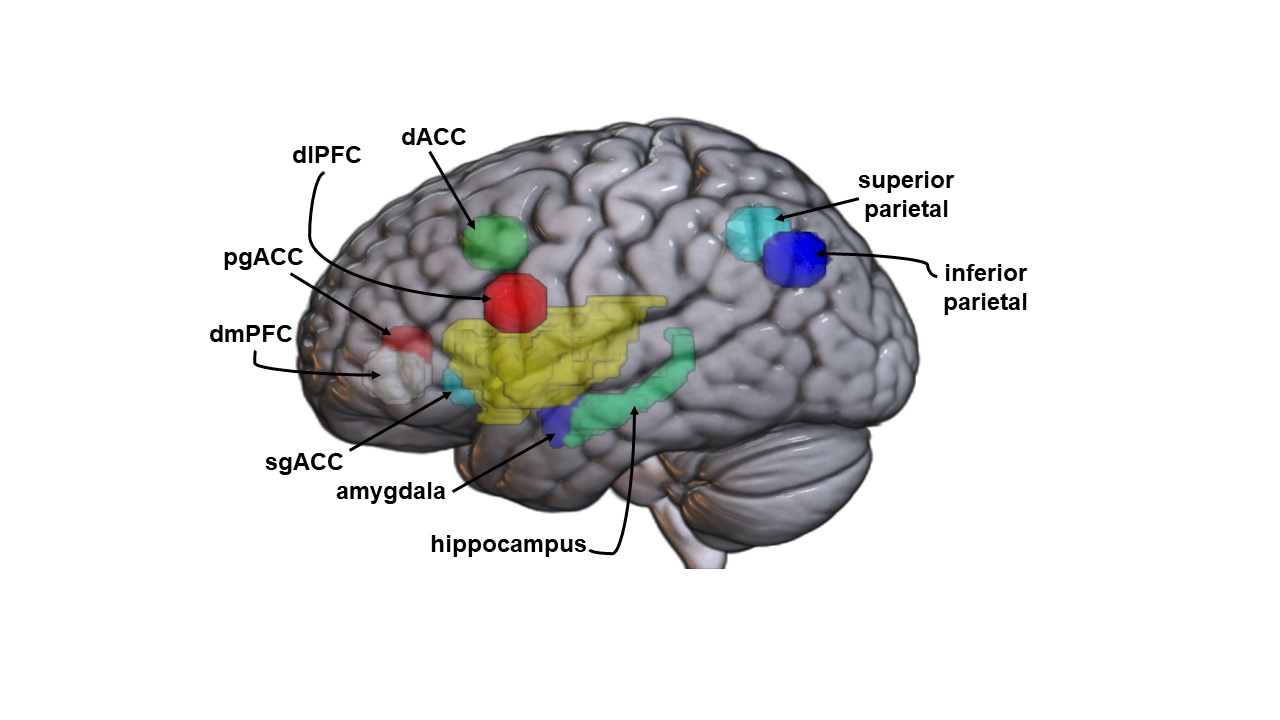
**

The insula region was further subdivided into the anterior and posterior insula based on Uddin et al. 2017; where the more anterior portion of the insula is typically comprised of the anterior, middle, and posterior short insular gyri, which are separated by the anterior and precentral insular sulcus and the posterior portion of the insula is comprised of the anterior and posterior long insular gyri, separated by the postcentral insular sulcus.

We used this definition to label the significant insula clusters into anterior vs. posterior insula. 10mm spheres centred on each significant cluster overlapping with the insula region (AAL atlas) was used for connectivity analyses.

**S.2 Comparisons between PTSD Responders, Non-responder and Controls at baseline and post-treatment.**

We assessed differences at pre and post-treatment relative to controls by splitting the PTSD group into treatment responders and non-responders (responders with >50% symptom reduction). We analysed only the significant effects observed and reported in the main manuscript document for this analysis.

We note that the sample size for this analysis is limited – 19 PTSD responders and 8 PTSD non-responders and hence these findings should be interpreted with caution.

At baseline, PTSD responders were similar to controls across all measures. PTSD non-responders, however, displayed significantly lower activation in the left hippocampus and right posterior insula relative to controls. At post-treatment, PTSD responders displayed lower activation in the right posterior insula relative to controls but were similar in all other areas. Non-responders demonstrated normalised activation at post-treatment across all regions.

For connectivity, both PTSD responders and non-responders were similar to controls across all measures at baseline and post-treatment.

Figures below show bar plots (mean+SE) at pre (T1) and post treatment (T2) for each PTSD subgroup and controls for the neural measures that were significantly associated with change in symptoms in the main analysis.

**Supplementary Figure S3: Changes in fMRI activation for treatment responders and non-responders for neural measures that were significantly associated with change in symptoms in the main analysis.**

**
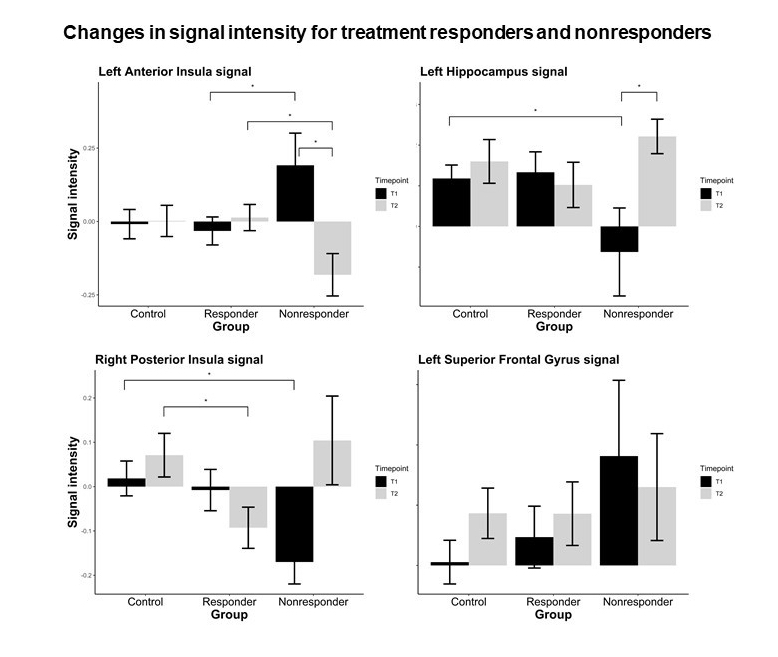
**

**Supplementary Table S2: Comparisons in activation between Responders, Non-Responders and Controls at the two timepoints.**

| **Comparison** | **t** | **p** | **Comparison** | **t** | **P** |
| --- | --- | --- | --- | --- | --- |
| *Left Anterior Insula* |  |  | *Left Hippocampus* |  |  |
| Responders vs controls at T1 | 0.335 | 0.74 | Responders vs controls at T1 | -0.261 | 0.796 |
| Nonresponders vs controls at T1 | -1.895 | 0.069 | Nonresponders vs controls at T1 | 2.16 | 0.04* |
| Responders vs nonresponders at T1 | -2.201 | 0.038* | Responders vs nonresponders at T1 | 1.882 | 0.07 |
| Responders vs controls at T2 | -0.161 | 0.873 | Responders vs controls at T2 | 0.746 | 0.46 |
| Nonresponders vs controls at T2 | 1.874 | 0.078 | Nonresponders vs controls at T2 | -0.645 | 0.525 |
| Responders vs nonresponders at T2 | 2.305 | 0.031* | Responders vs nonresponders at T2 | -1.269 | 0.217 |
| T1 vs T2 for responders | -0.636 | 0.533 | T1 vs T2 for responders | 0.455 | 0.654 |
| T1 vs T2 for nonresponders | 3.496 | 0.013* | T1 vs T2 for nonresponders | -2.565 | 0.043* |
| *Right Posterior Insula* |  |  | *Left dlPFC* |  |  |
| Responders vs controls at T1 | 0.433 | 0.667 | Responders vs controls at T1 | -0.669 | 0.507 |
| Nonresponders vs controls at T1 | 2.582 | 0.016* | Nonresponders vs controls at T1 | -1.857 | 0.075 |
| Responders vs nonresponders at T1 | 1.993 | 0.058 | Responders vs nonresponders at T1 | -1.19 | 0.245 |
| Responders vs controls at T2 | 2.405 | 0.021* | Responders vs controls at T2 | 0.01 | 0.992 |
| Nonresponders vs controls at T2 | -0.327 | 0.746 | Nonresponders vs controls at T2 | -0.496 | 0.623 |
| Responders vs nonresponders at T2 | -2.04 | 0.053 | Responders vs nonresponders at T2 | -0.437 | 0.666 |
| T1 vs T2 for responders | 1.347 | 0.196 | T1 vs T2 for responders | -0.697 | 0.495 |
| T1 vs T2 for nonresponders | -2.412 | 0.052 | T1 vs T2 for nonresponders | 0.481 | 0.647 |

**Supplementary Figure S4: Changes in fMRI connectivity for treatment responders and non-responders for neural measures that were significantly associated with change in symptoms in the main analysis.**

**
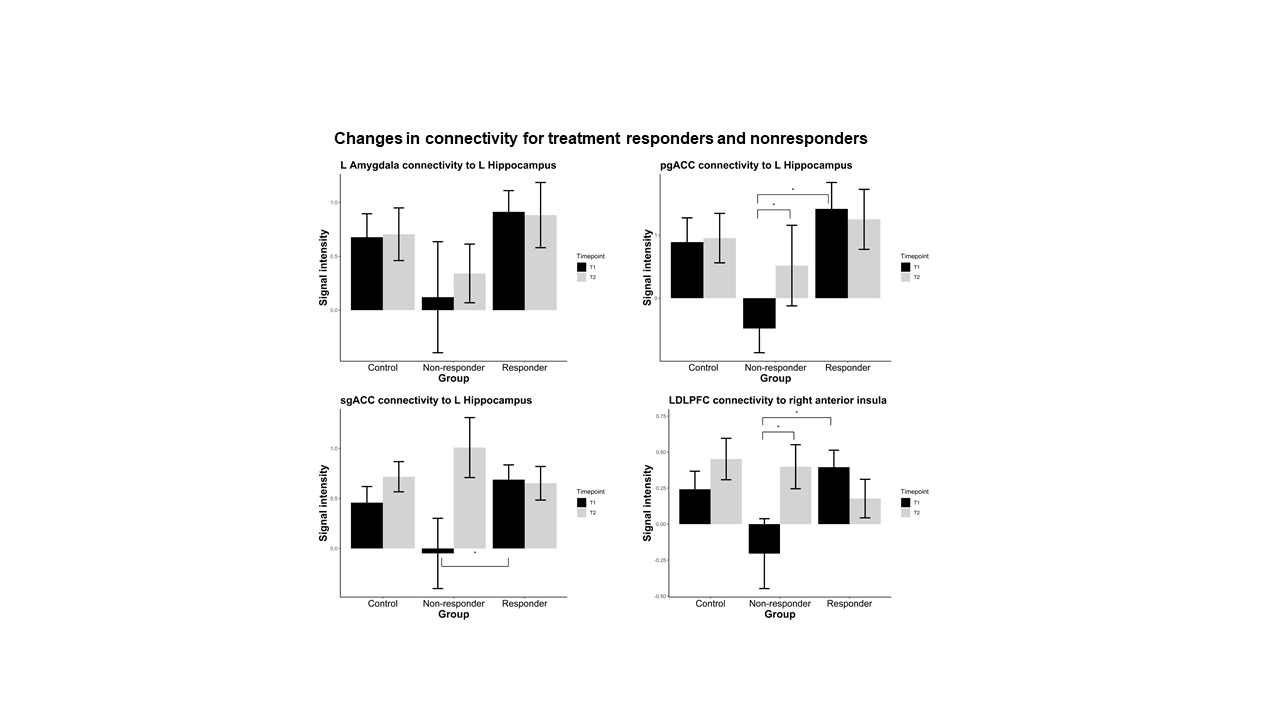
**

**Supplementary Table S3: Comparisons in connectivity between Responders, Non-Responders and Controls at the two timepoints.**

| **Comparison** | **t** | **p** | **Comparison** | **t** | **p** |
| --- | --- | --- | --- | --- | --- |
| *Left hippocampus connectivity to left amygdala* | | | *Left hippocampus connectivity to pgACC* | |  |
| Responders vs controls at T1 | -0.775 | 0.444 | Responders vs controls at T1 | -0.922 | 0.363 |
| Nonresponders vs controls at T1 | 1.139 | 0.265 | Nonresponders vs controls at T1 | 1.814 | 0.082 |
| Responders vs nonresponders at T1 | -1.778 | 0.091 | Responders vs nonresponders at T1 | -2.6 | 0.017* |
| Responders vs controls at T2 | -0.462 | 0.647 | Responders vs controls at T2 | -0.486 | 0.63 |
| Nonresponders vs controls at T2 | 0.752 | 0.459 | Nonresponders vs controls at T2 | 0.537 | 0.596 |
| Responders vs nonresponders at T2 | -1.029 | 0.316 | Responders vs nonresponders at T2 | -0.839 | 0.411 |
| T1 vs T2 for responders | 0.087 | 0.932 | T1 vs T2 for responders | 0.29 | 0.776 |
| T1 vs T2 for nonresponders | -0.461 | 0.664 | T1 vs T2 for nonresponders | -3.174 | 0.025* |
| *Left hippocampus connectivity to sgACC* | |  | *Right anterior insula connectivity to left dlPFC* | | |
| Responders vs controls at T1 | -1.02 | 0.315 | Responders vs controls at T1 | -0.865 | 0.393 |
| Nonresponders vs controls at T1 | 1.434 | 0.164 | Nonresponders vs controls at T1 | 1.667 | 0.108 |
| Responders vs nonresponders at T1 | -2.306 | 0.032* | Responders vs nonresponders at T1 | -2.481 | 0.022* |
| Responders vs controls at T2 | 0.289 | 0.775 | Responders vs controls at T2 | 1.358 | 0.183 |
| Nonresponders vs controls at T2 | -0.889 | 0.377 | Nonresponders vs controls at T2 | 0.189 | 0.852 |
| Responders vs nonresponders at T2 | 1.083 | 0.292 | Responders vs nonresponders at T2 | 0.922 | 0.367 |
| T1 vs T2 for responders | 0.175 | 0.863 | T1 vs T2 for responders | 1.421 | 0.176 |
| T1 vs T2 for nonresponders | -2.281 | 0.071 | T1 vs T2 for nonresponders | -3.626 | 0.015* |

**References:**

Berboth, S., & Morawetz, C. (2021). Amygdala-prefrontal connectivity during emotion regulation: A meta-analysis of psychophysiological interactions. *Neuropsychologia*, 153, 107767.

Buhle, J. T., Silvers, J. A., Wager, T. D., Lopez, R., Onyemekwu, C., Kober, H., ... & Ochsner, K. N. (2014). Cognitive reappraisal of emotion: a meta-analysis of human neuroimaging studies. *Cerebral cortex*, 24(11), 2981-2990.

Bryant, R. A., Erlinger, M., Felmingham, K., Klimova, A., Williams, L. M., Malhi, G., . . . Korgaonkar, M. S. (2020). Reappraisal-related neural predictors of treatment response to cognitive behavior therapy for post-traumatic stress disorder. *Psychol Med*, 1-11. doi:10.1017/S0033291720001129

Bryant, R. A., Erlinger, M., Felmingham, K., Malhi, G. S., O'Donnell, M. L., Williams, L. M., & Korgaonkar, M. S. (2020). Differential neural predictors of treatment response for fear and dysphoric features of posttraumatic stress disorder. *Depress Anxiety*. doi:10.1002/da.23061

Bryant, R. A., Felmingham, K., Kemp, A., Das, P., Hughes, G., Peduto, A., & Williams, L. (2008). Amygdala and ventral anterior cingulate activation predicts treatment response to cognitive behaviour therapy for post-traumatic stress disorder. *Psychol Med, 38*(4), 555-561. doi:S0033291707002231 [pii]

10.1017/S0033291707002231

Goldin, P. R., McRae, K., Ramel, W., & Gross, J. J. (2008). The neural bases of emotion regulation: reappraisal and suppression of negative emotion. *Biol Psychiatry, 63*(6), 577-586. doi:S0006-3223(07)00592-6 [pii]

10.1016/j.biopsych.2007.05.031

Kober, H., Barrett, L. F., Joseph, J., Bliss-Moreau, E., Lindquist, K., & Wager, T. D. (2008). Functional grouping and cortical–subcortical interactions in emotion: a meta-analysis of neuroimaging studies. *Neuroimage*, 42(2), 998-1031.

Korgaonkar, M. S., Erlinger, M., Breukelaar, I. A., Boyce, P., Hazell, P., Antees, C., . . . Malhi, G. S. (2019). Amygdala Activation and Connectivity to Emotional Processing Distinguishes Asymptomatic Patients With Bipolar Disorders and Unipolar Depression. *Biol Psychiatry Cogn Neurosci Neuroimaging, 4*(4), 361-370. doi:10.1016/j.bpsc.2018.08.012

Korgaonkar, M. S., Grieve, S. M., Etkin, A., Koslow, S. H., & Williams, L. M. (2013). Using standardized fMRI protocols to identify patterns of prefrontal circuit dysregulation that are common and specific to cognitive and emotional tasks in major depressive disorder: first wave results from the iSPOT-D study. *Neuropsychopharmacology, 38*(5), 863-871. doi:10.1038/npp.2012.252

Lang, P. J., Bradley, M.M., Cuthbert, B.N. . (2005). *International affective picture system (IAPS): affective ratings of pictures and instruction manual*. Retrieved from Gainesville, FL::

Niendam, T. A., Laird, A. R., Ray, K. L., Dean, Y. M., Glahn, D. C., & Carter, C. S. (2012). Meta-analytic evidence for a superordinate cognitive control network subserving diverse executive functions. *Cognitive, Affective, & Behavioral Neuroscience*, 12(2), 241-268.

Ochsner, K. N., Bunge, S. A., Gross, J. J., & Gabrieli, J. D. (2002). Rethinking feelings: an FMRI study of the cognitive regulation of emotion. *J Cogn Neurosci, 14*(8), 1215-1229. doi:10.1162/089892902760807212

Rai, S., Griffiths, K., Breukelaar, I. A., Barreiros, A. R., Chen, W., Boyce, P., . . . Korgaonkar, M. S. (2021). Investigating neural circuits of emotion regulation to distinguish euthymic patients with bipolar disorder and major depressive disorder. *Bipolar Disord, 23*(3), 284-294. doi:10.1111/bdi.13042

Uddin LQ, Nomi JS, Hébert-Seropian B, Ghaziri J, Boucher O. Structure and Function of the Human Insula. J Clin Neurophysiol. 2017 Jul;34(4):300-306. doi: 10.1097/WNP.0000000000000377. PMID: 28644199; PMCID: PMC6032992

Williams, L. M., Korgaonkar, M. S., Song, Y. C., Paton, R., Eagles, S., Goldstein-Piekarski, A., . . . Etkin, A. (2015). Amygdala Reactivity to Emotional Faces in the Prediction of General and Medication-Specific Responses to Antidepressant Treatment in the Randomized iSPOT-D Trial. *Neuropsychopharmacology, 40*(10), 2398-2408. doi:10.1038/npp.2015.89
